# *O*-GlcNAcylation-induced GSK-3β activation deteriorates pressure overload-induced heart failure *via* lack of compensatory cardiac hypertrophy in mice

**DOI:** 10.3389/fendo.2023.1122125

**Published:** 2023-03-22

**Authors:** Mahito Matsuno, Shunichi Yokoe, Takehiro Nagatsuka, Hirofumi Morihara, Kazumasa Moriwaki, Michio Asahi

**Affiliations:** ^1^ Department of Pharmacology, Faculty of Medicine, Osaka Medical and Pharmaceutical University, Osaka, Japan; ^2^ Center for Medical Research & Development, Osaka Medical and Pharmaceutical University, Osaka, Japan

**Keywords:** O-GlcNAcylation, heart failure, hypertrophy, transverse aortic constriction (TAC), GSK-3β

## Abstract

*O*-GlcNAc transferase (OGT) modulates many functions of proteins *via O*-GlcNAcylation that adds *O*-linked β-*N*-acetylglucosamine (*O*-GlcNAc) to the serine/threonine residues of proteins. However, the role of *O*-GlcNAcylation in cardiac remodeling and function is not fully understood. To examine the effect of *O*-GlcNAcylation on pressure overload-induced cardiac hypertrophy and subsequent heart failure, transverse aortic constriction (TAC) surgery was performed in wild type (WT) and *Ogt* transgenic (*Ogt*-Tg) mice. Four weeks after TAC (TAC4W), the heart function of *Ogt*-Tg mice was significantly lower than that of WT mice (reduced fractional shortening and increased ANP levels). The myocardium of left ventricle (LV) in *Ogt*-Tg mice became much thinner than that in WT mice. Moreover, compared to the heart tissues of WT mice, *O*-GlcNAcylation of GSK-3β at Ser9 was increased and phosphorylation of GSK-3β at Ser9 was reduced in the heart tissues of *Ogt*-Tg mice, resulting in its activation and subsequent inactivation of nuclear factor of activated T cell (NFAT) activity. Finally, the thinned LV wall and reduced cardiac function induced by TAC4W in *Ogt*-Tg mice was reversed by the treatment of a GSK-3β inhibitor, TDZD-8. These results imply that augmented *O*-GlcNAcylation exacerbates pressure overload-induced heart failure due to a lack of compensatory cardiac hypertrophy *via O*-GlcNAcylation of GSK-3β, which deprives the phosphorylation site of GSK-3β to constantly inactivate NFAT activity to prevent cardiac hypertrophy. Our findings may provide a new therapeutic strategy for cardiac hypertrophy and subsequent heart failure.

## 1 Introduction

*O*-GlcNAcylation, a post-translational modification of serine/threonine protein residues by *O*-linked β-*N*-acetylglucosamine (*O*-GlcNAc), is a dynamic and reversible process regulating many cellular functions including cell cycle regulation, metabolism, protein synthesis, epigenetic signaling, and calcium handling (1, 2). In the heart, it has been reported that *O*-GlcNAcylation play important roles in cardiac hypertrophy or heart failure (3, 4). Although *O*-GlcNAcylation is upregulated during cardiac hypertrophy and subsequent heart failure (5, 6), the regulatory mechanism of *O*-GlcNAcylation on the cardiac pathologies is not fully understood.

The addition and removal of *O*-GlcNAc on serine/threonine residues of proteins are catalyzed by *O*-GlcNAc transferase (OGT) and *O*-GlcNAcase (OGA) (7). OGT adds UDP-GlcNAc to hydroxy groups in the serine/threonine residues of many proteins and is known to mediate many cellular processes such as immunity (8) and cell cycle (9), and is also implicated in the pathological process of diseases such as diabetes (10) and cancer (9). Although OGT often competes with a serine/threonine phosphokinase to catalyze the *O*-GlcNAcation of the protein instead of phosphorylation, the interplay between *O*-GlcNAcation and phosphorylation varies; *O*-GlcNAcation sometimes promotes phosphorylation (11).

Left ventricular pressure overload caused by stresses such as hypertension and aortic stenosis evoke myocardial hypertrophy. It is widely believed that cardiac hypertrophy is an important intermediate stage in the progression process of heart failure. Sustained cardiac hypertrophy causes several harmful effects such as the development of heart failure and sudden death (12–14); however, it is also thought to be a compensational process for maintaining normal cardiac function (15). During the intermediate stage, the heart can circulate enough blood volume throughout the body by hypertrophic myocardium; however, if the stress continues to be loaded, it can cause dilated cardiomyopathy, followed by heart failure (16).

Many cellular signaling pathways regulate the hypertrophic response of cardiomyocytes (17). For example, the nuclear factor of activated T cells (NFAT) is known to transcribe several genes that are involved in cardiac hypertrophy. When NFAT is dephosphorylated by calcineurin, this makes it translocate from the cytosol to the nucleus and transcribes hypertrophic genes (18–20). On the contrary, when NFAT is phosphorylated by glycogen synthase kinase-3β (GSK-3β), this makes it translocate from the nucleus to the cytosol and the signaling pathways of cardiac hypertrophy are inhibited (21). It has been reported that transgenic mice overexpressing activated calcineurin in the heart showed enhanced cardiac hypertrophy and rapid progression to heart failure (18). The GSK-3β is known to be phosphorylated at Ser9 by Akt, and phosphorylation inhibits the activity of GSK-3β (22–24). GSK-3β is active when the external signals are absent; however, once the hypertrophic signal such as endothelin-1 (ET-1), isoproterenol, and aortic banding stimulate cardiomyocytes, GSK-3β is phosphorylated and its activity is inhibited (25). A previous study has shown that the overexpression of constitutively active GSK-3β in cultured cardiomyocytes attenuate cardiac hypertrophy induced by hypertrophic agents such as ET-1 and phenylephrine by blocking the NFAT nuclear translocation (25). Transgenic mice overexpressing a constitutively active form of GSK-3β were shown to reduce the cardiac hypertrophy induced by the activation of calcineurin, β-adrenergic stimulation, and pressure overload (26). It has also been reported that the inactivation of GSK-3β with lithium promotes pressure overload-induced cardiac hypertrophy in rats *via* β-catenin (27). These studies clearly show that NFAT is one of the most important molecules for inducing cardiac hypertrophy and GSK-3β is a critical negative regulator of cardiac hypertrophy signaling pathways. *O*-GlcNAcylation plays several roles in the impairment of cardiac function *via* modification of several of the proteins involved in progression to heart failure (3, 28, 29). In the present study, we used *Ogt* transgenic (*Ogt*-Tg) mice and induced heart failure by transverse aortic constriction (TAC) surgery to investigate the role of *O*-GlcNAcylation in cardiac hypertrophy. TAC surgery has been used to examine pressure overload hypertrophy and heart failure *in vivo* worldwide (30). Using the TAC method, we revealed that the heart function and wall thickness of *Ogt*-Tg mice were significantly lower than those of wild type (WT) mice. Moreover, we demonstrated that the inhibition of *O*-GlcNAcylation-induced activation of the GSK-3β signaling pathway improved cardiac remodeling in TAC-induced *Ogt*-Tg mice. Taken together, our results reveal the pivotal role of *O*-GlcNAcylation on the GSK-3β signaling pathway for cardiac dysfunction and remodeling by pressure overload.

## 2 Materials and methods

### 2.1 Antibodies and reagents

For Western blotting and immunohistochemistry, anti-OGT antibody (sc-32921), anti-BNP antibody (sc-67455), and anti-NFATc3 antibody (sc-8405) were purchased from Santa Cruz Biotechnology (Santa Cruz, CA, USA). Anti-*O*-GlcNAc antibody (MA1-072) was purchased from Affinity Thermo Scientific (Waltham, MA, USA). Anti-ANP (ab-91250) antibody was purchased from Abcam (Cambridge, MA, USA). Anti-phospho-GSK-3β, anti-GSK-3β, anti-phospho-NF-κB, anti-NF-κB, anti-phospho-Smad3, anti-Smad3, and anti-NFAT antibodies were purchased from Cell Signaling Technology (Danvers, MA, USA). Anti-Collagen III antibody (BS1531) was purchased from Bioworld Technology (Bloomington, MN, USA). For cell culture and *in vivo* experiments, GSK-3β inhibitor, TDZD-8, was purchased from Tokyo Chemical Industry (Tokyo, Japan). Thiamet G (TMG) was purchased from Cayman Chemical (Ann Arbor, MI, USA). Angiotensin II (Ang II) was purchased from Sigma-Aldrich (St. Louis, MO, USA).

### 2.2 Animal experiments

All animal experiments were conducted under the guidelines for the care and use of animals approved by Osaka Medical and Pharmaceutical University (protocol #2020-087). We used WT and *Ogt*-Tg mice (C57BL/6J, male) that expresses *Ogt* under the control of the CAG promoter (31). The mice (10-12 week-old) were anesthetized with 2,2,2-tribromoethanol. TAC surgery was performed under a dissecting microscope, with a small animal respirator, at a rate of 110 cycles/min. Aortic constriction was performed by tying a 7-0 silk string ligature around a 26-gauge needle, and then removing the needle. Since it was difficult for *Ogt*-Tg mice to survive for 4 weeks under the pressure overload by normal TAC surgery, we reduced the severity of the TAC surgery in this study. A sham surgery was performed following the same surgical procedure without tying the silk suture for the control group. Echocardiography (Nemio30; Toshiba Medical Systems, Japan) was performed without anesthetics before surgery, and 4 weeks after surgery, with the following parameters: LV ejection fraction (EF), LV fractional shortening (FS), end-diastolic LV internal dimension (LVIDd), end-systolic LV internal dimention (LVIDs), and then the heart was excised from the mice after being euthanized for Western blot and histological analyses.

### 2.3 Western blot and immunoprecipitation

Hearts excised from mice 4 weeks after the TAC or sham surgery were homogenized in lysis buffer (50 mM HEPES (pH 7.4), 5 mM sodium pyrophosphate, 10 mM sodium fluoride, 1 mM sodium orthovanadate, 10 mM β-glycerophosphate, and 1 mM phenylmethylsulfonyl fluoride) containing a proteasome inhibitor cocktail (WAKO Pure Chemical Industries, Osaka, Japan). Homogenates were centrifuged at 4°C for 10 min at 10,000 rpm. Protein concentration was measured by the bicinchoninic acid assay method according to the manufacturer’s instructions. Supernatants were mixed with sodium dodecyl sulfate (SDS) sample buffer and boiled for 5 min. The boiled samples were cooled at room temperature (22–28°C) and subjected to SDS-polyacrylamide gel electrophoresis. Separated proteins were transferred to a PVDF membrane (MERK Millipore, Burlington, MA, USA). The membrane was incubated in tris-buffered saline (TBST) containing 5% skim milk at room temperature for 1 h. Subsequently, the membrane was incubated with primary antibody in TBST containing 5% skim milk at 4°C overnight. The membrane was washed in TBST for 10 min three times and then incubated with secondary antibody in TBST containing 5% skim milk for 1 h. The membrane was washed in TBST for 10 min three times and detection was performed using Luminata Crescendo Western HRP (MERK Millipore) and Fusion FX7 (Vilber-Luormat, Germany).

Co-immunoprecipitation was performed using Sure Beads Protein G Magnetic Beads (Bio-Rad, Hercules, CA, USA) according to the manufacturer’s instructions. The pull-downed eluates were used for Western blot analysis with the antibodies of interest.

### 2.4 Histological analyses

For histological analysis, hearts were arrested in diastole, fixed with 4% paraformaldehyde, embedded in paraffin. Paraffin-embedded sections were stained with Masson’s trichrome for the detection of collagen fibers.

### 2.5 GSK-3β inhibition in mice

One week after the TAC or sham surgery, mice were injected intraperitoneally daily with a GSK-3β inhibitor, TDZD-8 (10 mg/kg/day, i.p.), dissolved in dimethylsulfoxide (DMSO): phosphate-buffered saline (PBS); 1:10 for 3 weeks. When daily intraperitoneal injection ended, echocardiography was performed, then the hearts were excised from the mice for Western blot analysis, Masson’s trichrome staining, and immunofluorescence staining.

### 2.6 GSK-3β inhibition in H9c2 cells

Rat cardiomyoblast cells (H9c2 cells) were cultured in Dulbecco’s modified Eagle’s medium with 10% fetal bovine serum. After 48 h incubation with serum-free medium, the cells were treated with PBS (solvent control), 100 nM Ang II, or Ang II plus 20 μM TDZD-8 for 48 h. The cells were further treated with or without 5 μM TMG for 2 h.

### 2.7 Immunofluorescence staining

The paraffin-embedded LV sections and H9c2 cells were fixed with 4% paraformaldehyde for 10 min, followed by blocking and permeabilization with 10% bovine serum albumin and 0.1% Triton-X100 for 15 min. The LV sections and H9c2 cells were then incubated with primary antibody against NFATc3 (1:200) at room temperature overnight. After washing three times with PBS, they were incubated with secondary Alexa Fluor 488-conjugated goat anti-mouse IgG antibody (1:200) at room temperature for 1 h. After washing 3 times with PBS, they were mounted using Vectashield mounting medium (Vector Laboratories) with 4’ 6-diamidino-2-phenylindole (DAPI) and observed under a confocal laser microscope (SP8, Leica, Germany).

### 2.8 Statistical analyses

Differences between more than two groups were analyzed using a two-way analysis of variance (ANOVA) followed by the Tukey’s *post hoc* test. The significant differences between two groups were evaluated by the F-test followed by the Student’s t-test.

## 3 Results

### 3.1 Increased *O*-GlcNAcylation in the hearts of *Ogt*-Tg mice four weeks after TAC (TAC4W)

**Figure 1:**
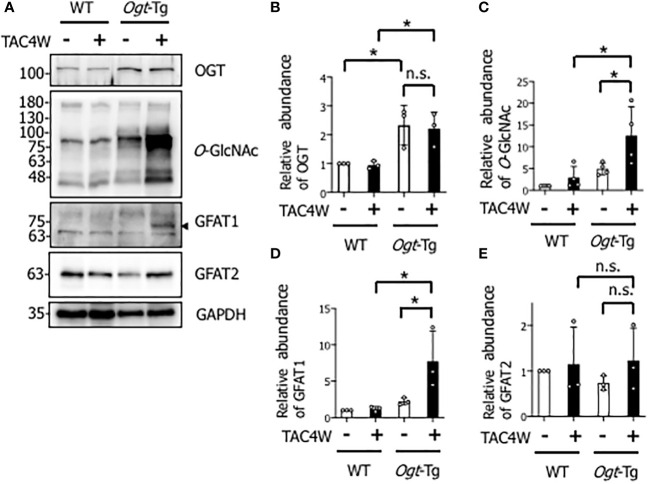
Increased O-GlcNAcylation in the hearts of Ogt-Tg mice four weeks after TAC (TAC4W). **(A)** Western blot analysis for OGT, O-GlcNAc, GFAT1, and GFAT2 in heart tissues from WT and Ogt-Tg mice with or without TAC4W. Representative data was designated. **(B–E)** Quantifications of OGT, O-GlcNAc, GFAT1, and GFAT2 levels in **(A)** from 3 independent experiments using ImageJ software. The data were evaluated by two-way analysis of variance (ANOVA) followed by Tukey’s test. Values are shown as mean ± SD (*P<0.05). n.s., not significant; TAC, transverse aortic constriction; GFAT, glutamine-fructose-6-phosphate transaminase.

To investigate the effect of OGT overexpression on pressure overload-induced cardiac hypertrophy or failure, TAC or sham surgery was performed in WT and *Ogt*-Tg mice. After TAC4W, *O*-GlcNAcylation was significantly increased in the hearts of *Ogt*-Tg mice, although the increase was not significant in those of WT mice, probably due to the less severity of the TAC surgery than normal (**Figures 1A, C**). There were no differences in the expression level of OGT in WT and *Ogt*-Tg mice after TAC4W (**Figures 1A, B**). To clarify the discrepancy between the rate of changes of *O*-GlcNAcylation and OGT expression levels in *Ogt*-Tg mice after TAC4W, we examined the expression level of glutamine-fructose-6-phosphate transaminase (GFAT) that generates UDP-*N*-acetylglucosamine (UDP-GlcNAc), the substrate for *O*-GlcNAcylation; the expression level of GFAT1 increased drastically in *Ogt*-Tg mice after TAC4W (**Figures 1A, D**), whereas GFAT2 expression was not significantly changed (**Figures 1A, E**). These data suggest that increased *O*-GlcNAcylation in the hearts of *Ogt*-Tg mice after TAC4W may be due to increased expression level of GFAT1, not GFAT2.

### 3.2 Reduced cardiac function in *Ogt*-Tg mice after TAC4W

**Figure 2:**
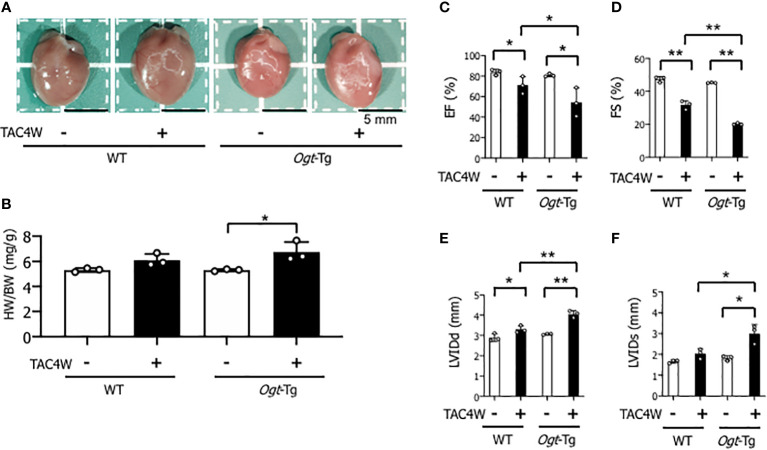
Reduced cardiac function in Ogt-Tg mice after TAC4W. **(A)** Hearts of representative WT and Ogt-Tg mice after TAC4W. Scale bars, 5mm. **(B)** Heart weight/body weight ratio (HW/BW, in mg/g) in TAC-treated WT and Ogt-Tg mice. The data were analyzed using two-way analysis of variance (ANOVA) followed by Tukey’s test. Values are shown as the mean ± SD value (n=3). *P<0.05. **(C–F)** Echocardiographic parameters of the mice; **(C)** EF, ejection fraction, **(D)** FS, fractional shortening, **(E)** LVIDd, end-diastolic left ventricular (LV) internal dimension, **(F)** LVIDs, end-systolic LV internal dimension. The data were evaluated by two-way analysis of variance (ANOVA), followed by Tukey’s test. **p<0.01. *p<0.05. n.s., not significant.

**Figure 3:**
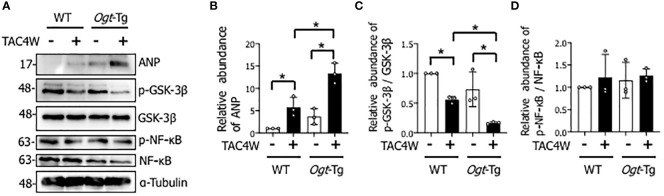
Decreased phosphorylation of GSK-3β in the hearts of Ogt-Tg mice after TAC4W. **(A)** Western blot analysis for ANP, GSK-3β phosphorylation, and NF-κB phosphorylation in heart tissues from WT and Ogt-Tg with or without TAC4W. Representative data was designated. **(B)** Quantifications of ANP levels in **(A)** from three independent experiments using ImageJ software, and evaluated by two-way analysis of variance (ANOVA) followed by Tukey’s test. Values are shown as mean ± SD (n=3). *P<0.05. **(C)** The ratios of p-GSK-3β/GSK-3β expression intensity were quantified using ImageJ software, and evaluated by two-way analysis of variance (ANOVA) followed by Tukey’s test. Values are shown as mean ± SD value (n=3). *P<0.05. **(D)** The ratios of p-NF-κB/NF-κB expression intensity were quantified using ImageJ software, and evaluated by two-way analysis of variance (ANOVA) followed by Tukey’s test. Values are shown as mean ± SD value (n=3). TAC, transverse aortic constriction; ANP, atrial natriuretic peptides.

Heart weight/body weight ratio was significantly increased in *Ogt*-Tg mice after TAC4W compared to sham group, whereas the increase was not significant in WT mice (**Figures 2A, B**). The echocardiography showed that EF and FS were significantly decreased and LVIDd was significantly increased in WT and *Ogt*-Tg mice after TAC4W. LVIDs was significantly increased in *Ogt*-Tg mice after TAC4W, but not in WT mice. The decrease rates in EF and FS and increase rates in LVIDd and LVIDs in *Ogt*-Tg mice after TAC4W were significantly higher compared to those in WT mice (**Figures 2C–F**). Myocardial ANP, a biochemical marker for left ventricular dysfunction, was significantly increased in *Ogt*-Tg mice after TAC4W (**Figures 3A, B**).

### 3.3 Decreased phosphorylation of GSK-3β in the hearts of *Ogt*-Tg mice after TAC4W

To investigate the molecular mechanisms underlying TAC-induced cardiac dysfunction in *Ogt*-Tg mice, we examined the GSK-3β and NF-κB signaling pathways that are involved in cardiac hypertrophy (21) (32). We found that the phosphorylation of GSK-3β, which reflects inactivation of the pathway, in the heart tissues of *Ogt*-Tg mice was significantly lower than those of WT mice after TAC4W (**Figures 3A, C**). In contrast, the phosphorylation of NF-κB, which reflects its activation of the pathway, was not significantly changed (**Figures 3A, D**). Collectively, the GSK-3β signaling pathway, but not NF-κB, may be involved in cardiac hypertrophy after TAC4W that induces cardiac dysfunction.

### 3.4 Restoration of compensatory cardiac hypertrophy and fibrosis by the treatment of TDZD-8 in *Ogt*-Tg mice after TAC4W

**Figure 4:**
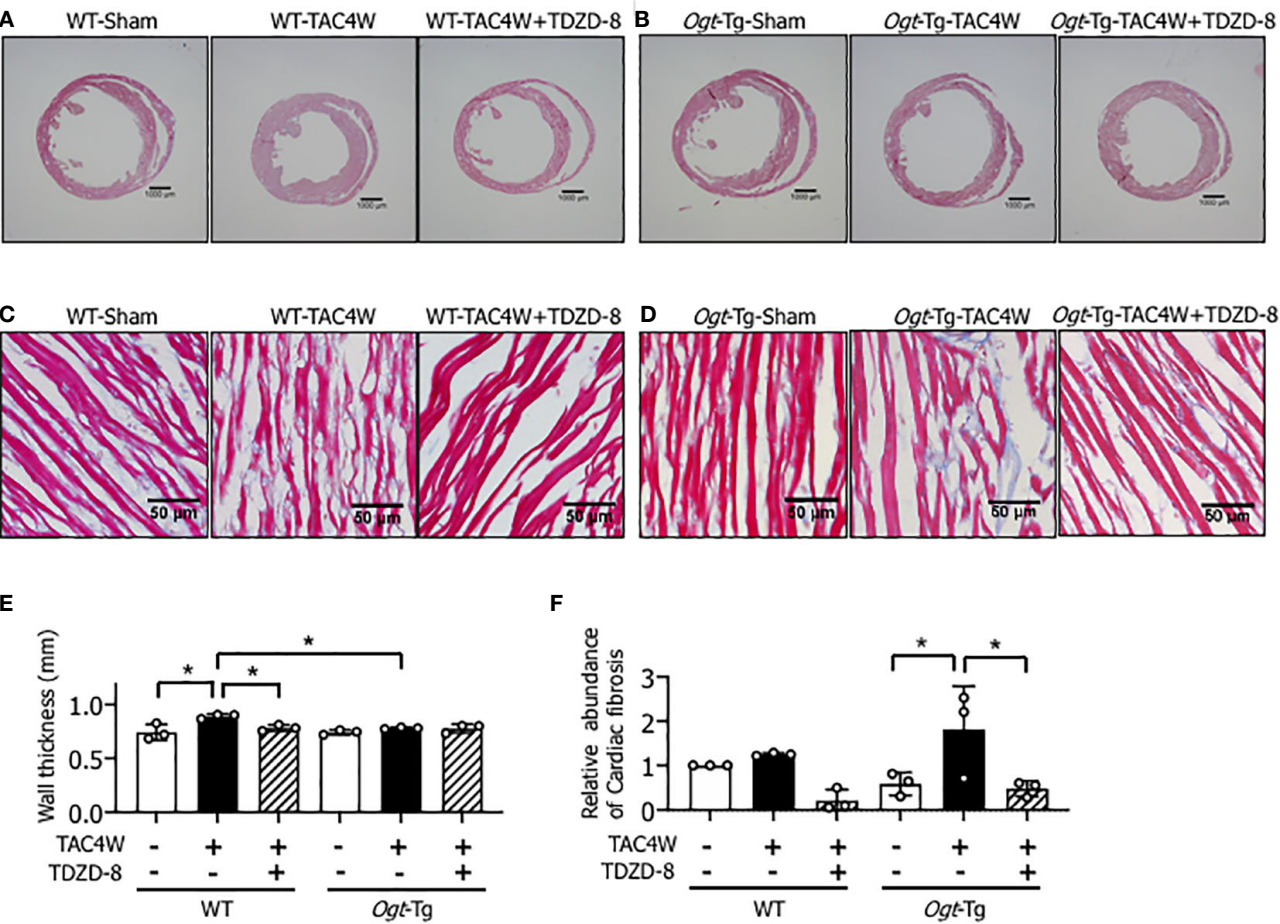
Restoration of compensatory cardiac hypertrophy and fibrosis by the treatment of TDZD-8 in Ogt-Tg mice after TAC4W. **(A–F)** Masson's trichrome staining in heart tissues from TAC4W-treated WT and Ogt-Tg mice with or without TDZD-8 (10 mg/kg/day, 3 weeks, IP). Scale bar: **(A, B)** = 1000 μm, **(C, D)** = 50 μm. **(E)** Quantitative analysis of interventricular wall thickness evaluated by Masson's trichrome staining presented in **(A–D)**. The data were evaluated by two-way analysis of variance (ANOVA) followed by Tukey’s test. Values are shown as mean±SD (n=3). *P<0.05. **(F)** Quantitative analysis of cardiac fibrosis evaluated by Masson's trichrome staining presented in **(A–D)**. The data were evaluated by two-way analysis of variance (ANOVA) followed by Tukey’s test. Values are shown as mean±SD (n=3). *P<0.05. TAC, transverse aortic constriction.

Four weeks after the TAC (TAC4W) or sham surgery in WT and *Ogt*-Tg mice, morphological changes in the hearts from both mice groups was evaluated. The macroscopic images of Masson’s trichrome staining showed that the myocardium in *Ogt*-Tg mice did not show hypertrophy, whereas the LV dimension was enlarged compared to WT mice (**Figures 4A, B**). We found that TDZD-8 improved the TAC-induced hypertrophy in *Ogt*-Tg mice, indicating that the GSK-3β signaling pathway was involved in this morphology (**Figure 4B**). Moreover, increased LV wall thickness observed in WT mice after TAC4W was restored with TDZD-8 treatment (**Figures 4A, E**). In microscopic images, cardiac fibrosis was significantly increased in *Ogt*-Tg mice after TAC4W compared to sham mice, and TDZD-8 reduced the cardiac fibrosis in *Ogt*-Tg mice (**Figures 4D, F**), whereas it was not significantly changed in WT mice after TAC4W compared to sham mice, probably due to the less severity of the TAC surgery than normal (**Figures 4C, F**).

### 3.5 Restoration of cardiac systolic dysfunction and enlargement by the treatment of TDZD-8 in WT and *Ogt*-Tg mice after TAC4W

**Figure 5:**
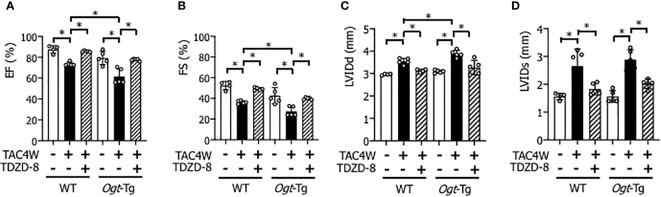
Restoration of cardiac dysfunction and enlargement by the treatment of TDZD-8 in WT and Ogt-Tg mice after TAC4W. Echocardiographic analysis of **(A)** Ejection fraction (EF), **(B)** Fractional shortening (FS), **(C)** left ventricular (LV) end-diastolic dimension (LVIDd), **(D)**end-systolic LV internal dimension (LVIDs) in TAC4W-treated WT and Ogt-Tg mice. The data were evaluated by two-way analysis of variance (ANOVA) followed by Tukey’s test. Values are shown as mean ± SD (n=4). *P<0.05. TAC, transverse aortic constriction.

The echocardiography showed that EF and FS were decreased and LVIDd and LVIDs were increased in WT and *Ogt*-Tg mice after TAC4W, and the changes of cardiac parameters were all restored by the treatment of TDZD-8 (**Figures 5A–D**).

### 3.6 Restoration of decreased phosphorylation of GSK-3β by the treatment of TDZD-8 in the hearts of *Ogt*-Tg mice after TAC4W

**Figure 6:**
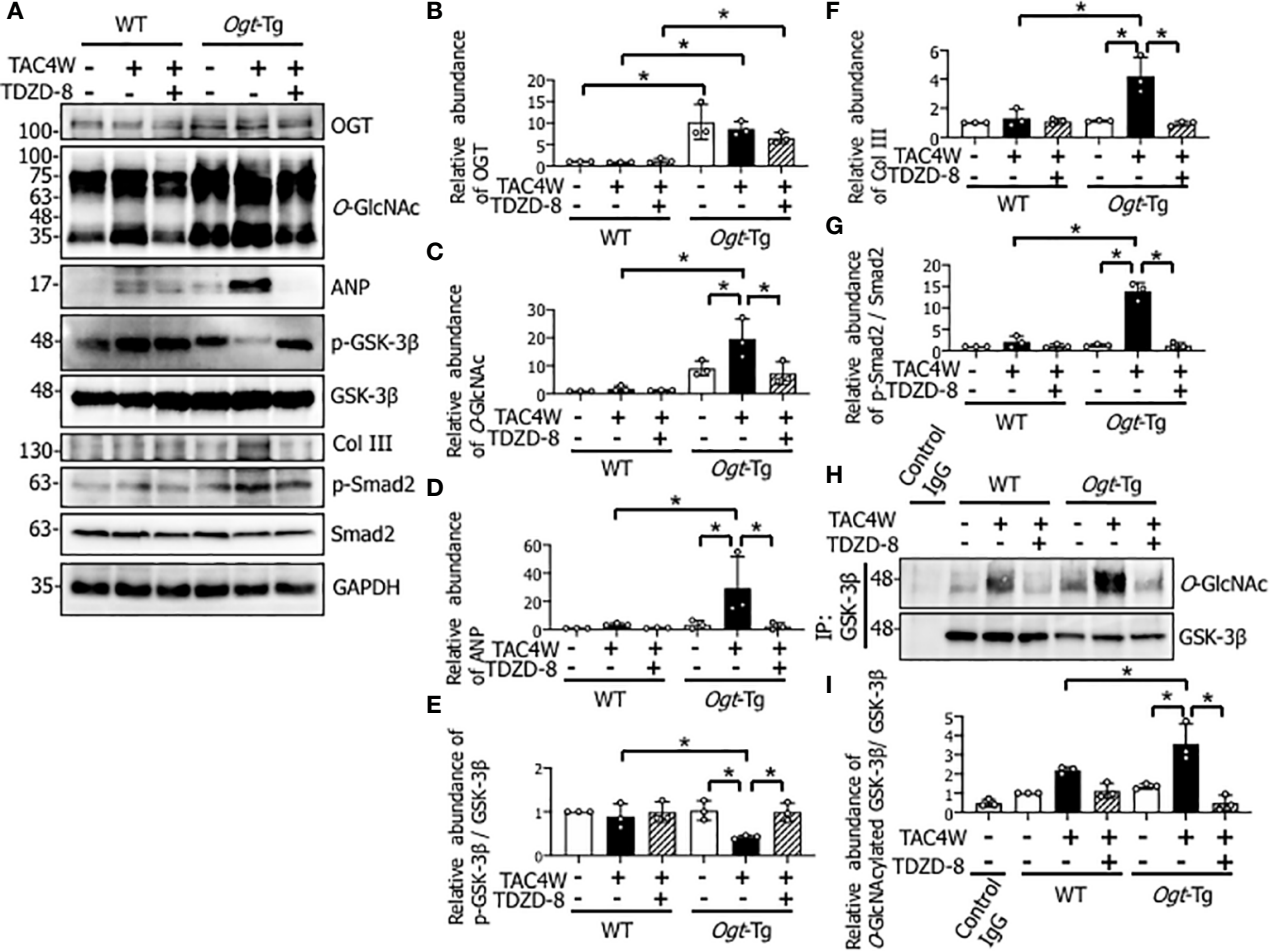
Restoration of decreased phosphorylation of GSK-3β by the treatment of TDZD-8 in the hearts of Ogt-Tg mice after TAC4W. **(A)** Western blot analysis for OGT, O-GlcNAc, ANP, Col III, and phosphorylation of GSK-3β, Smad2 in heart tissues from TAC4W-induced WT and Ogt-Tg mice with or without GSK-3β inhibitor (TDZD-8 (10 mg/kg/day, 3 weeks, IP)) treatment. Representative data was designated. **(B–G)** Quantifications of OGT, O-GlcNAc, ANP, Col III, and the ratios of p-GSK-3β/GSK-3β, p-Smad2/Smad2 expression intensity in **(A)** from three independent experiments using ImageJ software. The data were evaluated by two-way analysis of variance (ANOVA) followed by Tukey’s test. Values are shown as mean ± SD. *P<0.05. **(H)** Immunoprecipitation for O-GlcNAcylated GSK-3β in heart tissues from TAC4W-induced WT and Ogt-Tg mice with or without GSK-3β inhibitor (TDZD-8 (10 mg/kg/day, 3 weeks, IP)) treatment. Representative data was designated. **(I)** The intensity of each band for the O-GlcNAcylated GSK-3β expression (n=3) was measured using ImageJ software, and evaluated by two-way analysis of variance (ANOVA) followed by Tukey’s test. Values are shown as mean ± SD (n=3). *P<0.05. TAC, transverse aortic constriction; ANP, atrial natriuretic peptides; Col III, collagen type III.

Since cardiac fibrosis was significantly increased in *Ogt*-Tg mice after TAC4W and the increase was restored with the treatment of TDZD-8 (**Figure 4**), we examined the effect of the TAC surgery and TDZD-8 treatment on the Smad signaling pathway related to fibrosis and the expression of collagen III that is the main constituent of the interstitial matrix to form fibrosis. The result showed that the phosphorylation level of Smad2 and expression level of Collagen III were both increased in *Ogt*-Tg mice after TAC4W, and the increase was restored with the treatment of TDZD-8 (**Figures 6A, F, G**). The TAC surgery significantly reduced GSK-3β phosphorylation in the hearts of *Ogt*-Tg mice, and the reduced phosphorylation was restored by the treatment of TDZD-8 (**Figures 6A, E**). Given that total *O*-GlcNAcylation level was increased in the *Ogt*-Tg mice and the level was synergistically increased after TAC4W (**Figures 1A, C**), we examined whether *O*-GlcNAcylation was involved in the reduction of the GSK-3β signaling pathway. Co-immunoprecipitation study showed that the levels of *O*-GlcNAcylated GSK-3β in the heart tissues of *Ogt*-Tg mice was significantly increased after TAC4W, which was restored by the treatment of TDZD-8 (**Figures 6H, I**). *O*-GlcNAcylation generally competes with phosphorylation of target proteins (11, 33); therefore, these results imply that increased *O*-GlcNAcylation of GSK-3β in *Ogt*-Tg mice after TAC4W may result in the reduction of phosphorylated GSK-3β and the restoration of the GSK-3β phosphorylation *via* reduced GSK-3β *O*-GlcNAcylation by the treatment of TDZD-8. *O*-GlcNAcylation (**Figures 6A, C**) and myocardial ANP (**Figures 6A, D**) were significantly increased in *Ogt*-Tg mice after TAC4W, and the increase was restored with the treatment of TDZD-8. There were no differences in the expression level of OGT in WT and *Ogt*-Tg mice after TAC4W with or without the treatment of TDZD-8 (**Figures 6A, B**).

### 3.7 Inactivation of the NFAT signaling pathway with TMG treatment and the restoration by the treatment of TDZD-8 in Ang II-stimulated H9c2 cells

**Figure 7:**
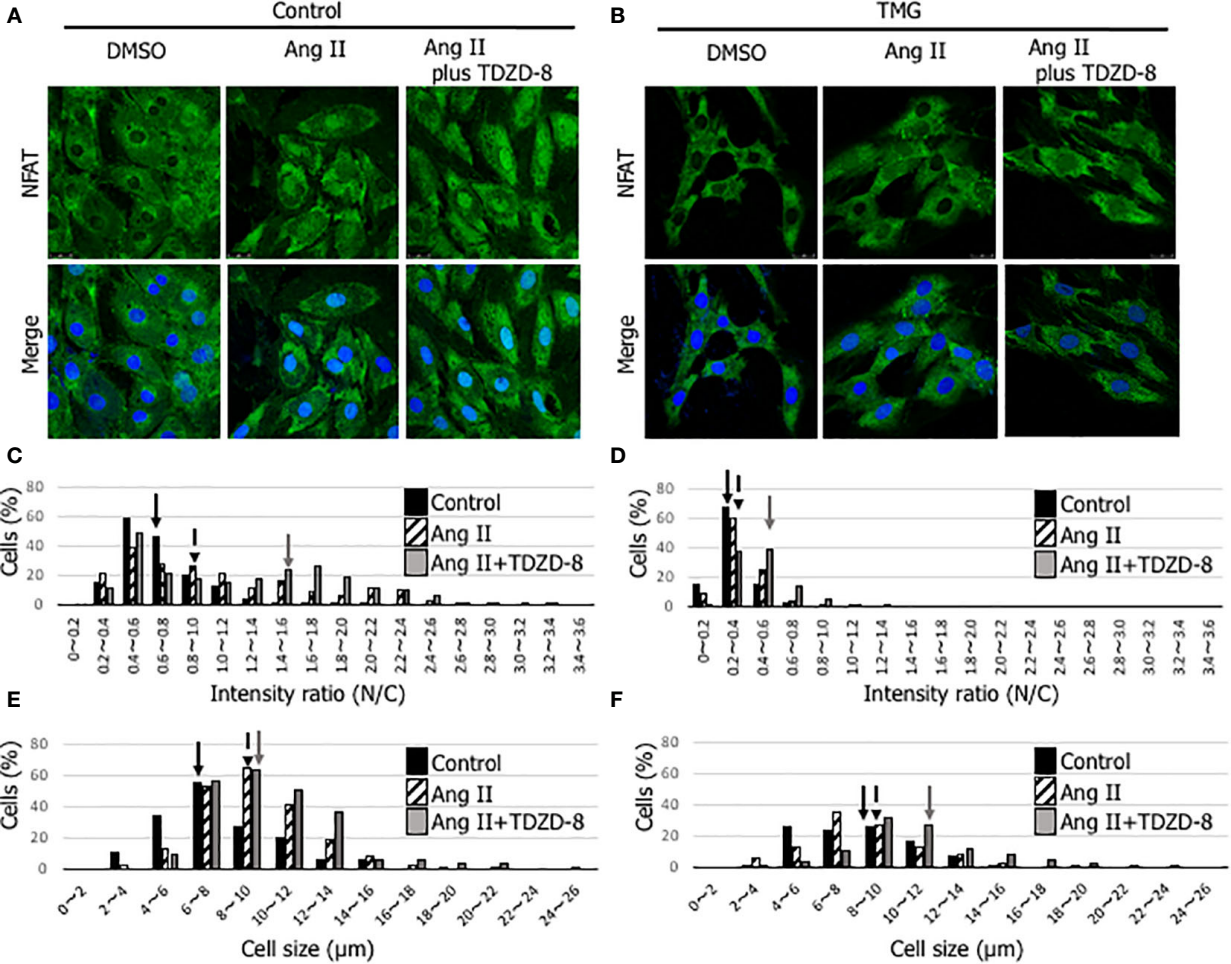
Inactivation of the NFAT signaling pathway with TMG treatment and the restoration by the treatment of TDZD-8 in Ang II-stimulated H9c2 cells. **(A, B)** NFAT (green) and DAPI (blue) staining was performed to examine the effect of DMSO- **(A)** or TMG- (5 μM) **(B)** treatment with or without the pretreatment of TDZD-8 (2 μM) on angiotensin II (1 μM)-stimulated H9c2 cells. Representative data was designated. The cells were fixed with 4% PFA, and observed with confocal microscopy. Scale bar: 25 µm. **(C, D)** The nuclear/cytosol (N/C) ratio was calculated by ImageJ software to examine the effect of DMSO- **(C)** or TMG- **(D)** treatment with or without the pretreatment of TDZD-8 in Ang II-stimulated H9c2 cells, and designated in histogram. **(E, F)** The cell size was measured to examine the effect of DMSO- **(E)** or TMG- **(F)** treatment with or without the pretreatment of TDZD-8 in Ang II-stimulated H9c2 cells, and designated in histogram. Closed bar, Control cells; Hatched bar, Ang II-stimulated cells; Shaded bar, Ang II-stimulated cells with the pretreatment of TDZD-8. Arrows indicate median values of the distributions. TMG,Thiamet G; DMSO, Solvent control.

NFAT is a master transcription factor that regulates genes involved in cardiac hypertrophy. Phosphorylated NFAT by GSK-3β is retained in the cytoplasm and cannot induce cardiac hypertrophy, indicating that the phosphorylation state of GSK-3β is important for the NFAT signaling pathway (16). To examine whether TMG treatment, which increases *O*-GlcNAcylation, affects subcellular localization of NFAT, we employed an Ang II-induced cardiomyocyte hypertrophic model with H9c2 cells. As shown in **Figures 7A–D**, nuclear translocation of NFAT was observed with Ang II stimulation in dimethyl sulfoxide (DMSO) (solvent control)-treated H9c2 cells, whereas it was not observed in TMG-treated H9c2 cells. The translocation was accelerated by the treatment of TDZD-8 in DMSO-treated H9c2 cells (**Figures 7A, C**), whereas it was not changed by the treatment of TDZD-8 in TMG-treated H9c2 cells (**Figures 7B, D**), which is consistent with the data showing that the treatment of TDZD-8 restored cardiac function and cardiac hypertrophy in *Ogt*-Tg mice after TAC4W (**Figures 4**, **5**). NFAT is one of the major promotors of cardiac hypertrophy; therefore, the cell size was measured in Ang II-stimulated H9c2 cells in the presence or absence of TMG or TDZD-8. The cell size in H9c2 cells was increased with Ang II stimulation regardless of TDZD-8 addition, whereas TMG treatment minimized the effect, which was associated with nuclear translocation of NFAT (**Figures 7E, F**). Collectively, the activation of GSK-3β by *O*-GlcNAcylation prevents pressure overload and Ang II-induced cardiac hypertrophy by inhibiting the NFAT signaling pathway.

## 4 Discussion

Many factors such as hypertension, valvular disease, ischemic heart disease, and arrhythmia cause heart failure. When heart failure is caused by excess afterload due to hypertension and aortic valve stenosis, cardiac hypertrophy occurs in the intermediate stage of heart failure. In general, cardiac hypertrophy is thought to be harmful and maladaptive because it may cause arrhythmia and cardiac arrest. The first stage of cardiac hypertrophy, however, maintains myocardial contraction and is also thought to be compensational (34).

In *Ogt*-Tg mice, cardiac dysfunction with lack of hypertrophy was observed after TAC4W (**Figure 4**). To clarify the mechanism by which cardiac hypertrophy did not occur in *Ogt*-Tg mice after TAC4W, Western blot and immunoprecipitation analyses were performed to examine the activities of NF-κB and GSK-3β, which are major regulators of cardiac hypertrophy and *O*-GlcNAcylated proteins (21, 32). The results showed that GSK-3β phosphorylation was lower and reciprocally the *O*-GlcNAcylation was higher in the heart tissues of *Ogt*-Tg mice than those of the WT mice (**Figures 3A, C**, **6H, I**). The activated form of NF-κB, NF-κB p65 phosphorylation, was not significantly changed (**Figures 3A, D**). It is known that phosphorylation at Ser9 of GSK-3β inhibits its activity (35–38). To investigate how the inhibition of GSK-3β phosphorylation by *O*-GlcNAcylation affects cardiac hypertrophy and function *in vivo*, we injected TDZD-8, in *Ogt*-Tg mice before TAC. TDZD-8 restored cardiac enlargement and dysfunction in *Ogt*-Tg mice after TAC4W (**Figures 4**, **5**).

GSK-3β induces cardiac hypertrophy through the promotion of NFAT nuclear translocation; therefore, we confirmed that the TDZD-8 treatment induced the nuclear translocation of NFAT in Ang II-stimulated H9c2 cells (**Figure 7**). The results demonstrate that augmented *O*-GlcNAcylation by OGT overexpression deprives the phosphorylation site of GSK-3β probably by the addition of *O*-GlcNAc to the Ser9 residue, resulting in the activation of GSK-3β to phosphorylate NFAT. The stable activation of GSK-3β by augmented *O*-GlcNAcylation is likely to induce heart failure *via* the lack of cardiac hypertrophy in *Ogt*-Tg mice after TAC4W. It is thought that the observed cardiac hypertrophy was compensatory rather than maladaptive because cardiac function of *Ogt*-Tg mice was severely reduced after TAC4W, although cardiac hypertrophy with normal function was observed in the heart tissues of WT mice in the pressure overload. Given that the lack of cardiac hypertrophy was observed in the heart tissues of *Ogt*-Tg mice, it is conceivable that the inhibition of NFAT *via* GSK-3β by *O*-GlcNAcylation aggravated the pressure overload-induced heart failure after TAC4W.

How augmented *O*-GlcNAcylation affects cardiac hypertrophy is a controversial topic (39, 40). The effects of augmented *O*-GlcNAcylation caused by stress or disease on the heart is complex and highly dependent on the specific context of these events, such as acute or chronic heart failure. *O*-GlcNAcylation was augmented in c-Myc transgenic mice where cardiac hypertrophy was induced (41), whereas elevated *O*-GlcNAcylation after TAC was blunted in c-Myc knockout mice where cardiac hypertrophy was attenuated (42). c-Myc is known to be *O*-GlcNAcylated at Thr58, which is also phosphorylated (43). When phosphorylated at Thr58, c-Myc is degraded. Most recently, it is reported that nucleotide sugar transporters, SLC35B4 contribute to c-Myc stabilization by modifying its *O*-GlcNAcylation in hepatocellular carcinoma (44). Therefore, the c-Myc *O*-GlcNAcylation might be one of the effectors for the cardiac hypertrophy after pressure overload. c-Myc *O*-GlcNAcylation may inhibit its phosphorylation and stabilize its protein level to keep its transcriptional activity, followed by induction of cardiac hypertrophy. Conversely, another study showed that cardiomyocyte-specific OGT knockout mice induced fibrotic, apoptotic, and hypertrophic hearts, and only 12% of them survived to weaning age (45). Recently, Umapathi et al. reported that excessive *O*-GlcNAcylation leads to heart failure and premature death due to mitochondrial energy deficit in *Ogt*-Tg mice (4). On the other hand, our *Ogt*-Tg mice in the present study does not indicate premature death, or sudden death with or without TAC surgery. We assume that OGT expression level in our *Ogt*-Tg mice could be lower than that in the mice established by Umapathi et al. possibly due to the difference of transgene copy number or insertion locus. The variety of phenotype is useful to comprehend different characteristics of *O*-GlcNAcylation during heart failure, providing a better understanding of the pathophysiology of the disease.

The present study supports the inhibition of cardiac hypertrophy by augmented *O*-GlcNAcylation. Our data shows that the activation of GSK-3β by *O*-GlcNAcylation inhibits compensatory hypertrophy *via* inactivation of NFAT. Given that TDZD-8 can reverse cardiac hypertrophy and subsequent dysfunction after TAC4W (**Figures 4**–**6**), the change in GSK-3β activity by its *O*-GlcNAcylation could be strongly involved in the cardiac hypertrophy and dysfunction, although it is conceivable that there are other cardiac hypertrophy-related regulators that can be activated by *O*-GlcNAcylation.

Increased OGT expression induced by infection of adenovirus prolonged calcium transient decays and significantly decreased cardiac type sarco/endoplasmic reticulum Ca^2+^-ATPase (SERCA2a) protein levels (46). Phospholamban (PLN), a major regulator of SERCA2a, is known to be *O*-GlcNAcylated and its *O*-GlcNAcylation has been shown to be implicated in reduced cardiac function (47). *O*-GlcNAcylation of myofilaments attenuates Ca^2+^ sensitivity, which is restored by decreasing *O*-GlcNAcylation of myofilaments (48). These studies indicate that the enhancement of *O*-GlcNAcylation impairs both cardiac relaxation and contraction. Our data suggests that sustained enhancement of *O*-GlcNAcylation impairs cardiac compensatory hypertrophy and contraction 4 weeks after pressure overload. There were no significant phenotypes in *Ogt*-Tg mice without pressure overload; however, cardiac function in *Ogt*-Tg mice was lower than that in WT mice after TAC4W. This may be because of a lack of compensatory hypertrophy. In another words, *Ogt*-Tg mice are likely to be vulnerable to pressure overload. Because *O*-GlcNAcylation is persistently augmented in patients with diabetes, the pathophysiology of *Ogt*-Tg mice after TAC4W may be resemble to that of patients with diabetes who have a complication of hypertension. Those patients may be more likely to have heart failure *via* attenuation of compensatory cardiac hypertrophy. In conclusion, we showed that augmented *O*-GlcNAcylation exacerbates pressure overload-induced heart failure due to a lack of compensatory cardiac hypertrophy *via O*-GlcNAcylation of GSK-3β, which deprives the phosphorylation site of GSK-3β to constantly inactivate NFAT activity to prevent cardiac hypertrophy. Our findings may provide a new therapeutic strategy for cardiac hypertrophy and subsequent heart failure.

## Data availability statement

The original contributions presented in the study are included in the article/supplementary material. Further inquiries can be directed to the corresponding author.

## Ethics statement

The animal study was reviewed and approved by the ethics committee of Osaka Medical and Pharmaceutical University.

## Author contributions

SY, MA contributed to study design. MM, SY, TN performed animal experiments. MM, SY performed cell culture and histology analyses. MM, SY, HM, KM, MA analyzed data. MM, SY, MA drafted the manuscript. MA performed study supervision. All authors contributed to the article and approved the submitted version.

## References

[1] Dias WB Cheung WD Wang Z Hart GW. Regulation of calcium/calmodulin-dependent kinase IV by O-GlcNAc modification. J Biol Chem (2009) 284:21327–37. doi: 10.1074/jbc.M109.007310 PMC2755857 19506079

[2] Love DC Krause MW Hanover JA. O-GlcNAc cycling: Emerging roles in development and epigenetics. Semin Cell Dev Biol (2010) 21:646–54. doi: 10.1016/j.semcdb.2010.05.001 PMC2917487 20488252

[3] Zhu WZ El-Nachef D Yang X Ledee D Olson AK. O-GlcNAc transferase promotes compensated cardiac function and protein kinase a O-GlcNAcylation during early and established pathological hypertrophy from pressure overload. J Am Heart Assoc (2019) 8:e011260. doi: 10.1161/JAHA.118.011260 31131693 PMC6585351

[4] Umapathi P Mesubi OO Banerjee PS Abrol N Wang Q Luczak ED. Excessive O-GlcNAcylation causes heart failure and sudden death. Circulation (2021) 143:1687–703. doi: 10.1161/CIRCULATIONAHA.120.051911 PMC8085112 33593071

[5] Dassanayaka S Brainard RE Watson LJ Long BW Brittian KR DeMartino AM. Cardiomyocyte ogt limits ventricular dysfunction in mice following pressure overload without affecting hypertrophy. Basic Res Cardiol (2017) 112:23. doi: 10.1007/s00395-017-0612-7 28299467 PMC5555162

[6] Facundo HT Brainard RE Watson LJ Ngoh GA Hamid T Prabhu SD. O-GlcNAc signaling is essential for NFAT-mediated transcriptional reprogramming during cardiomyocyte hypertrophy. Am J Physiol Heart Circ Physiol (2012) 302:H2122–30. doi: 10.1152/ajpheart.00775.2011 PMC3362113 22408028

[7] Stephen HM Adams TM Wells L. Regulating the regulators: Mechanisms of substrate selection of the O-GlcNAc cycling enzymes OGT and OGA. Glycobiology (2021) 31:724–33. doi: 10.1093/glycob/cwab005 PMC8351506 33498085

[8] Wu JL Chiang MF Hsu PH Tsai DY Hung KH Wang YH. O-GlcNAcylation is required for b cell homeostasis and antibody responses. Nat Commun (2017) 8:1854. doi: 10.1038/s41467-017-01677-z 29187734 PMC5707376

[9] Jiang M Qiu Z Zhang S Fan X Cai X Xu B. Elevated O-GlcNAcylation promotes gastric cancer cells proliferation by modulating cell cycle related proteins and ERK 1/2 signaling. Oncotarget (2016) 7:61390–402. doi: 10.18632/oncotarget.11359 PMC5308659 27542217

[10] Park K Saudek CD Hart GW. Increased expression of beta-n-acetylglucosaminidase in erythrocytes from individuals with pre-diabetes and diabetes. Diabetes (2010) 59:1845–50. doi: 10.2337/db09-1086 PMC2889787 20413512

[11] Wang Z Gucek M Hart GW. Cross-talk between GlcNAcylation and phosphorylation: Site-specific phosphorylation dynamics in response to globally elevated O-GlcNAc. Proc Natl Acad Sci USA (2008) 105:13793–8. doi: 10.1073/pnas.0806216105 PMC2544533 18779572

[12] Levy D Garrison RJ Savage DD Kannel WB Castelli WP. Prognostic implications of echocardiographically determined left ventricular mass in the framingham heart study. N Engl J Med (1990) 322:1561–6. doi: 10.1056/NEJM199005313222203 2139921

[13] Schiattarella GG Hill JA. Inhibition of hypertrophy is a good therapeutic strategy in ventricular pressure overload. Circulation (2015) 131:1435–47. doi: 10.1161/CIRCULATIONAHA.115.013894 PMC4408778 25901069

[14] Hunter JJ Chien KR. Signaling pathways for cardiac hypertrophy and failure. N Engl J Med (1999) 341:1276–83. doi: 10.1056/NEJM199910213411706 10528039

[15] Takano H Zou Y Akazawa H Toko H Mizukami M Hasegawa H. Inhibitory molecules in signal transduction pathways of cardiac hypertrophy. Hypertens Res (2002) 25:491–8. doi: 10.1291/hypres.25.491 12358132

[16] Hill JA Olson EN. Cardiac plasticity. N Engl J Med (2008) 358:1370–80. doi: 10.1056/NEJMra072139 18367740

[17] Akazawa H Komuro I. Roles of cardiac transcription factors in cardiac hypertrophy. Circ Res (2003) 92:1079–88. doi: 10.1161/01.RES.0000072977.86706.23 12775656

[18] Molkentin JD Lu JR Antos CL Markham B Richardson J Robbins J. A calcineurin-dependent transcriptional pathway for cardiac hypertrophy. Cell (1998) 93:215–28. doi: 10.1016/S0092-8674(00)81573-1 PMC4459646 9568714

[19] Wilkins BJ Molkentin JD. Calcium-calcineurin signaling in the regulation of cardiac hypertrophy. Biochem Biophys Res Commun (2004) 322:1178–91. doi: 10.1016/j.bbrc.2004.07.121 15336966

[20] Molkentin JD. Calcineurin-NFAT signaling regulates the cardiac hypertrophic response in coordination with the MAPKs. Cardiovasc Res (2004) 63:467–75. doi: 10.1016/j.cardiores.2004.01.021 15276472

[21] Tateishi A Matsushita M Asai T Masuda Z Kuriyama M Kanki K. Effect of inhibition of glycogen synthase kinase-3 on cardiac hypertrophy during acute pressure overload. Gen Thorac Cardiovasc Surg (2010) 58:265–70. doi: 10.1007/s11748-009-0505-2 20549454

[22] Cross DA Alessi DR Cohen P Andjelkovich M Hemmings BA. Inhibition of glycogen synthase kinase-3 by insulin mediated by protein kinase b. Nature (1995) 378:785–9. doi: 10.1038/378785a0 8524413

[23] Stambolic V Woodgett JR. Mitogen inactivation of glycogen synthase kinase-3 beta in intact cells *via* serine 9 phosphorylation. Biochem J (1994) 303(Pt 3):701–4. doi: 10.1042/bj3030701 PMC1137602 7980435

[24] Dajani R Fraser E Roe SM Young N Good V Dale TC. Crystal structure of glycogen synthase kinase 3 beta: structural basis for phosphate-primed substrate specificity and autoinhibition. Cell (2001) 105:721–32. doi: 10.1016/S0092-8674(01)00374-9 11440715

[25] Haq S Choukroun G Kang ZB Ranu H Matsui T Rosenzweig A. Glycogen synthase kinase-3beta is a negative regulator of cardiomyocyte hypertrophy. J Cell Biol (2000) 151:117–30. doi: 10.1083/jcb.151.1.117 PMC2189812 11018058

[26] Antos CL McKinsey TA Frey N Kutschke W McAnally J Shelton JM. Activated glycogen synthase-3 beta suppresses cardiac hypertrophy *in vivo* . Proc Natl Acad Sci USA (2002) 99:907–12. doi: 10.1073/pnas.231619298 PMC117404 11782539

[27] Yamamoto F Yamamoto H. Effect of inhibition of glycogen synthase kinase-3 on cardiac hypertrophy during acute pressure overload. Gen Thorac Cardiovasc Surg (2010) 58:263–4. doi: 10.1007/s11748-009-0562-6 20549453

[28] Peng ML Fu Y Wu CW Zhang Y Ren H Zhou SS. Signaling pathways related to oxidative stress in diabetic cardiomyopathy. Front Endocrinol (Lausanne) (2022) 13:907757. doi: 10.3389/fendo.2022.907757 35784531 PMC9240190

[29] Prakoso D Lim SY Erickson JR Wallace RS Lees JG Tate M. Fine-tuning the cardiac O-GlcNAcylation regulatory enzymes governs the functional and structural phenotype of the diabetic heart. Cardiovasc Res (2022) 118:212–25. doi: 10.1093/cvr/cvab043 33576380

[30] Yoo J Chepurko V Hajjar RJ Jeong D. Conventional method of transverse aortic constriction in mice. Methods Mol Biol (2018) 1816:183–93. doi: 10.1007/978-1-4939-8597-5_14 29987820

[31] Moriwaki K Asahi M. Augmented TME O-GlcNAcylation promotes tumor proliferation through the inhibition of p38 MAPK. Mol Cancer Res (2017) 15:1287–98. doi: 10.1158/1541-7786.MCR-16-0499 28536142

[32] Li Y Ha T Gao X Kelley J Williams DL Browder IW. NF-kappaB activation is required for the development of cardiac hypertrophy *in vivo* . Am J Physiol Heart Circ Physiol (2004) 283:H1712–20. doi: 10.1152/ajpheart.00124.2004 15142841

[33] Rani L Mallajosyula SS. Phosphorylation versus O-GlcNAcylation: Computational insights into the differential influences of the two competitive post-translational modifications. J Phys Chem B (2017) 121:10618–38. doi: 10.1021/acs.jpcb.7b08790 29077417

[34] Brancaccio M Fratta L Notte A Hirsch E Poulet R Guazzone S. Melusin, a muscle-specific integrin beta1-interacting protein, is required to prevent cardiac failure in response to chronic pressure overload. Nat Med (2003) 9:68–75. doi: 10.1038/nm805 12496958

[35] Frame S Cohen P Biondi RM. A common phosphate binding site explains the unique substrate specificity of GSK3 and its inactivation by phosphorylation. Mol Cell (2001) 7:1321–7. doi: 10.1016/S1097-2765(01)00253-2 11430833

[36] Cohen P Frame S. The renaissance of GSK3. Nat Rev Mol Cell Biol (2001) 2:769–76. doi: 10.1038/35096075 11584304

[37] de Sousa RT Zanetti MV Talib LL Serpa MH Chaim TM Carvalho AF. Lithium increases platelet serine-9 phosphorylated GSK-3beta levels in drug-free bipolar disorder during depressive episodes. J Psychiatr Res (2015) 62:78–83. doi: 10.1016/j.jpsychires.2015.01.016 25691093

[38] Grabinski T Kanaan NM. Novel non-phosphorylated serine 9/21 GSK3beta/alpha antibodies: Expanding the tools for studying GSK3 regulation. Front Mol Neurosci (2016) 9:123. doi: 10.3389/fnmol.2016.00123 27909397 PMC5112268

[39] Wright JN Collins HE Wende AR Chatham JC. O-GlcNAcylation and cardiovascular disease. Biochem Soc Trans (2017) 45:545–53. doi: 10.1042/BST20160164 PMC5640322 28408494

[40] Dos Passos Junior RR Bomfim GF Giachini FR Tostes RC Lima VV. O-Linked beta-N-Acetylglucosamine modification: Linking hypertension and the immune system. Front Immunol (2022) 13:852115. doi: 10.3389/fimmu.2022.852115 35371030 PMC8967968

[41] Olson AK Ledee D Iwamoto K Kajimoto M O'Kelly Priddy C Isern N. C-myc induced compensated cardiac hypertrophy increases free fatty acid utilization for the citric acid cycle. J Mol Cell Cardiol (2013) 55:156–64. doi: 10.1016/j.yjmcc.2012.07.005 PMC3524362 22828478

[42] Ledee D Smith L Bruce M Kajimoto M Isern N Portman MA. C-myc alters substrate utilization and O-GlcNAc protein posttranslational modifications without altering cardiac function during early aortic constriction. PloS One (2015) 10:e0135262. doi: 10.1371/journal.pone.0135262 26266538 PMC4534195

[43] Chou TY Hart GW Dang CV. C-myc is glycosylated at threonine 58, a known phosphorylation site and a mutational hot spot in lymphomas. J Biol Chem (1995) 270:18961–5. doi: 10.1074/jbc.270.32.18961 7642555

[44] Jiang T Yang J Yang H Chen W Ji K Xu Y. SLC35B4 stabilizes c-MYC protein by O-GlcNAcylation in HCC. Front Pharmacol (2022) 13:851089. doi: 10.3389/fphar.2022.851089 35308201 PMC8924407

[45] Watson LJ Long BW DeMartino AM Brittian KR Readnower RD Brainard RE. Cardiomyocyte ogt is essential for postnatal viability. Am J Physiol Heart Circ Physiol (2014) 306:H142–53. doi: 10.1152/ajpheart.00438.2013 PMC3920156 24186210

[46] Clark RJ McDonough PM Swanson E Trost SU Suzuki M Fukuda M. Diabetes and the accompanying hyperglycemia impairs cardiomyocyte calcium cycling through increased nuclear O-GlcNAcylation. J Biol Chem (2003) 278:44230–7. doi: 10.1074/jbc.M303810200 12941958

[47] Yokoe S Asahi M Takeda T Otsu K Taniguchi N Miyoshi E. Inhibition of phospholamban phosphorylation by O-GlcNAcylation: Implications for diabetic cardiomyopathy. Glycobiology (2010) 20:1217–26. doi: 10.1093/glycob/cwq071 20484118

[48] Ramirez-Correa GA Ma J Slawson C Zeidan Q Lugo-Fagundo NS Xu M. Removal of abnormal myofilament O-GlcNAcylation restores Ca2+ sensitivity in diabetic cardiac muscle. Diabetes (2015) 64:3573–87. doi: 10.2337/db14-1107 PMC4587639 26109417

